# Rethinking the tumor microbiome: Methodological challenges and a framework for rigorous research

**DOI:** 10.1002/mlf2.70107

**Published:** 2026-10-26

**Authors:** Lingxuan Zhu, Zhixing Deng, Weiming Mou, Gengwen Deng, Hongyu Lu, Liling Xu, Shujia Lin, Hongsen Huang, Keren Wu, Qiheng Liang, Hao Li, Ziwen Zheng, Anqi Lin, Peng Luo

**Affiliations:** ^1^ Department of Oncology, Zhujiang Hospital Southern Medical University Guangzhou China; ^2^ Department of Urology, Shanghai General Hospital Shanghai Jiao Tong University School of Medicine Shanghai China; ^3^ The First School of Clinical Medicine Southern Medical University Guangzhou China; ^4^ The Second School of Clinical Medicine Southern Medical University Guangzhou China; ^5^ Department of Microbiology, State Key Laboratory of Emerging Infectious Diseases, Carol Yu Centre for Infection, School of Clinical Medicine, Li Ka Shing Faculty of Medicine The University of Hong Kong Hong Kong China; ^6^ Shantou University Medical College Shantou China

**Keywords:** contamination, host depletion, intratumoral microbiome, low‐biomass samples

## Abstract

The intratumoral microbiome, as an essential component of the tumor microenvironment, has attracted increasing attention in recent years. The tumor microbiota displays an intricate interplay with its biological behaviors. However, emerging controversies, including the retraction of Poore et al.'s article, highlight significant challenges within this field. These issues extend beyond questions of reliability in this particular work to challenge fundamental theoretical assumptions and methodological validity across the entire field of tumor microbiome research, profoundly impacting its future direction. Here, we systematically summarize and discuss the current challenges and limitations in tumor microbiome research. We further propose a series of practical optimization strategies and solutions, from microbial quantification to functional experiments, providing a comprehensive framework covering the entire workflow of tumor microbiome analysis. With ongoing technical progress and standardized protocols, tumor microbiome research is poised to deliver novel insights into tumor biology and contribute to the development of improved therapeutic strategies.

## INTRODUCTION

While genetic mutations and epigenetic alterations are primary intrinsic drivers of tumor development, mounting evidence highlights microorganisms as crucial extrinsic factors^1^, ^2^. Historically, specific microorganisms (e.g., *Helicobacter pylori* and high‐risk human papillomavirus [HPV]) have been established as carcinogens that promote tumor development through mechanisms like chronic inflammation and host immunity suppression^3^, ^4^. Recently, multidimensional evidence—ranging from high‐throughput sequencing to direct visualization and functional validation—has challenged the historical dogma of sterile tumor tissues, revealing that tumors host diverse microbial communities^5^, ^6^.

However, analysis of microbial signals in host‐dominated, low‐biomass environments (such as tumor tissues) is fraught with signal‐to‐noise challenges^7^. In 2020, Poore et al. utilized large‐scale sequencing data from The Cancer Genome Atlas (TCGA) to systematically investigate tumor microbiomes across multiple cancer types^8^. This study rapidly accumulated broad academic influence, stimulating extensive re‐analysis of microbiome information and even accelerating early clinical translation. Nevertheless, subsequent independent analyses identified critical methodological flaws regarding sequence contamination, inadequate host depletion, and batch effect normalization^9^, ^10^. These massive false‐positive results and analytical errors ultimately led to the formal retraction of Poore et al.'s article by *Nature* in June 2024.

This retraction extends beyond a single study, and it highlights a systemic lack of standardized protocols and exposes fundamental methodological vulnerabilities across the broader tumor microbiome field. In this perspective, we systematically analyze the methodological challenges in tumor microbial quantification, particularly sequence contamination and data normalization. More importantly, we propose an optimized, comprehensive research framework covering the entire workflow, from rigorous sample collection and high‐throughput sequencing to functional experimental validation, aiming to provide crucial methodological guidance for this rapidly evolving field.

## MICROBIAL QUANTIFICATION PRINCIPLES USING WHOLE‐GENOME SEQUENCING (WGS) AND RNA SEQUENCING (RNA‐SEQ)

Unlike traditional microbial research, which often relies on specialized sequencing methods, such as 16S rRNA sequencing or metagenomic sequencing, Poore et al. used WGS and RNA‐seq data from TCGA to quantify microorganisms. This approach, representing a “culture‐independent” method for microbial detection, has a theoretical basis for quantifying microorganisms from data generated by high‐throughput sequencing of tumor tissue^11^. This feasibility stems from the unbiased nature of high‐throughput sequencing, which captures and sequences all genetic material present in the samples. For instance, during RNA sequencing of human tissue samples, all RNA sequences present are captured, including not only human RNA but also genetic material from microorganisms^12^. Taking an RNA‐seq dataset from a non‐small cell lung cancer biopsy sample (SRR17150697) stored in the National Center for Biotechnology Information (NCBI) database as an example, rapid taxonomic classification using the NCBI's SRA Taxonomy Analysis Tool (STAT) revealed that approximately 2.58% of sequences aligned with bacterial reference genomes, while less than 0.01% of reads were identified as fungi or archaea. This finding highlighted the complexity and diversity of microbial composition in the tumor microenvironment. Theoretically, after removing human sequences from the raw sequencing data (the “host depletion” process), non‐human reads containing microbial fragments can be obtained. Further alignment of these reads to microbial reference genomes allows for taxonomic classification and abundance estimation of microorganisms in the sample. This process also provides insights into the functional and metabolic characteristics of the microbial community present in the tumor^13^.

In fact, this analytical approach traces its origins to Meyerson et al., who introduced a method known as “computational subtraction” in 2002. This pioneering technique enabled comprehensive and unbiased microbial identification by removing from the library sequences highly similar to the human genome and subsequently aligning the remaining non‐human sequences against known pathogen databases. This breakthrough study laid the foundation for subsequent tumor microbiome analysis. Over time, this approach has been continuously optimized, leading to the development of advanced tools such as PathSeq and Kraken, which significantly improved the efficiency and accuracy of microbial sequence identification in sequencing data^11^, ^14^. These tools have established a framework for detecting microbial information in transcriptome and WGS data, which has been widely applied in numerous studies. This has further stimulated the scientific community's interest in mining microbial signals from existing sequencing data, thereby promoting the rapid development of tumor microbiome research^15^, ^16^.

Despite the controversies and limitations surrounding this quantification pipeline, it has still demonstrated accuracy and consistency with expected results in several key aspects, effectively detecting and quantifying tumor‐associated microorganisms in certain contexts^17^. HPV is a well‐established carcinogenic factor, with high‐risk HPV infection closely linked to the increasing incidence of head and neck squamous cell carcinoma (HNSC), while almost all cervical cancer cases can be attributed to high‐risk HPV infection^18^. Poore et al.'s study showed that when analyzing TCGA samples from patients clinically diagnosed with HPV‐positive cervical squamous cell carcinoma (CESC) or HNSC using the Kraken quantification method, significantly elevated HPV abundance was observed^8^. Ge et al.'s study further confirmed this finding, with HPV still being identified as the most abundant microorganism in cervical cancer samples even when using the latest reference genomes and optimized quantification workflows^19^. Similarly, in gastrointestinal tumor research, this method revealed significantly higher normalized abundance of *Fusobacterium* in colorectal cancer tumor samples compared to normal samples^19^, aligning with substantial evidence from previous studies that *Fusobacterium* is enriched in the tumor tissues and feces of colorectal cancer patients^16^. Moreover, Zhang et al. performed both WGS and quantitative PCR (qPCR) analyses on 27 gastric biopsy samples and found a strong positive correlation between WGS‐based *H. pylori* read counts and qPCR quantification of the *H. pylori*‐specific *ureA* gene^20^. Therefore, while this quantification pipeline has limitations and unresolved questions that require further investigation and refinement, it has shown utility in yielding accurate and biologically consistent results across multiple tumor microbiome studies. As such, it provides an important methodological foundation to build upon in this rapidly developing field of research.

## SEQUENCE CONTAMINATION AND METHODOLOGICAL FLAWS

Although microbial quantification based on WGS and RNA‐seq is theoretically feasible, Poore et al.'s study faced significant challenges due to two critical methodological flaws that ultimately led to its retraction. The first and most substantial issue stemmed from the inability to achieve effective host depletion, which resulted in extensive human sequence contamination within the microbial reads. This contamination gave rise to numerous false‐positive results, including the presence of unexplained taxa and abnormally high abundances of microorganisms that were inconsistent with biological expectations. The second major challenge involved the Voom–SNM normalization method. Specifically, prior information such as tumor types was unintentionally introduced into the data during the normalization process, and the machine learning models subsequently utilized these artificial signals, achieving unusually high classification accuracy for tumor identification. Additionally, the normalization process artificially changed zero values to non‐zero values, introducing features that did not actually exist, which further undermined the reliability of the analysis results^8^.

### Anomalous microbial reads arising from sequence contamination

Concerns about Poore et al.'s study initially focused on several unexpected microbial taxa. According to the analysis results, the microorganisms identified by machine learning algorithms as important predictors of tumor types showed biological profiles inconsistent with known human microbiota^9^. For example, in Poore et al.'s study, *Leucothrix* was identified as the most crucial bacterial genus in the machine learning model for distinguishing bladder cancer from other tumors. However, as *Leucothrix* is a bacterial genus that primarily inhabits marine macroalgae^21^, the probability of its presence in human tissues is exceedingly low. Similarly, in the machine learning model for identifying prostate cancer, *Ignicoccus*, a hyperthermophilic archaeal genus living in deep‐sea hydrothermal vents, was determined to be the most important feature. This finding has also been questioned, as *Ignicoccus*'s extreme thermophilic nature and specific metabolic requirements make it virtually impossible to survive in the human body^22^. The presence of these microorganisms in human tissues is considered biologically implausible. Therefore, the reliability of these microorganisms as features for tumor identification is highly questionable. The emergence of these anomalous taxa raises a crucial question: What led to these clearly unreasonable microbial quantification results?

#### Residual human sequences

In Poore et al.'s study, researchers extracted reads that did not align to the human reference genome from TCGA BAM files using SAMtools^23^. These reads were then matched against bacterial, archaeal, and viral reference genomes using the Kraken2 algorithm to obtain microbial abundance information^14^. However, because the majority of BAM files analyzed were aligned to hg19, an outdated human reference genome, numerous human sequences evaded detection and were erroneously misclassified as non‐human reads^24^. Gihawi et al.'s study confirmed this situation. Using Bowtie2, they realigned the “non‐human” reads from TCGA to the complete T2T–CHM13 human genome and found that approximately 51% of these reads in TCGA–Breast Invasive Carcinoma (BRCA) samples were actually reidentified as human sequences^10^, ^25^. Therefore, the supposedly human genome‐unmapped reads extracted by Poore et al. from BAM files still contained a large number of human sequences.

Moreover, the inclusion or exclusion of human genomes in the reference database used for Kraken microbial alignment significantly impacts the results. Notably, the standard Kraken database actually includes human reference genomes. It aims to create a reference database containing both microbial and human sequences, which can also help eliminate human sequence contamination during the classification process. However, Poore et al.'s study excluded the human genome in their own reference database, further increasing the proportion of human‐derived sequences being misassigned to microorganisms. Taking the TCGA–DK–A1AB sample from TCGA–Bladder Urothelial Carcinoma (BLCA) as an example, during microbial alignment, there was a striking difference in the number of reads assigned to the genus *Waddlia* when comparing databases with and without human genome sequences: The former yielded 1100‐fold more reads than the latter (197,811 vs. 174)^10^. Further alignment of the reads classified as *Waddlia* in the former case against the T2T–CHM13 human genome revealed that 98.9% of these reads matched human sequences. In summary, Poore et al.'s microbial quantification pipeline lacked adequate measures to prevent human‐derived sequence interference with microbial reads, such as the omission of an additional host depletion round and the exclusion of human reference genomes during microbial alignment. These measures could have minimized the contribution of human sequences.

#### Impact of reference genome quality on microbial quantification

In the process of microbial alignment, the quality of reference genome databases has a decisive impact on the accuracy of microbial quantification results^26^. In Poore et al.'s study, the microbial alignment process utilized a database containing thousands of incomplete (“draft”) bacterial genomes, which might have been contaminated with human sequences, thereby leading to microbial misidentification and overestimation of microbial abundance^27^. This issue highlights the importance of using high‐quality reference genomes in tumor microbiome research. For sample s2707, Poore et al.'s study identified 327,985 *Streptococcus* reads. However, when Gihawi et al. aligned the same sample against a database containing only complete bacterial genomes, the *Streptococcus* read count dropped to 1. This striking difference clearly demonstrates the substantial impact of reference genome quality on microbial quantification results. Beyond misclassification, the absolute quantity of intratumoral bacteria remains a subject of debate. When researchers applied rigorous filtering criteria, excluding reads aligning to the complete human T2T–CHM13 genome, they found that the actual microbial signal in many TCGA tumor samples was extremely low, often indistinguishable from noise or contamination^9^. This suggests that while intratumoral bacteria likely exist, their abundance may be much lower than previously estimated by WGS/RNA‐seq mining, highlighting the inherent pitfalls of using low‐biomass host‐dominated sequencing data for microbiome analysis without rigorous controls.

In another study exploring the intratumoral fungal ecology, contamination in microbial reference genomes also led to severe bias in quantification results. Narunsky‐Haziza et al. identified 54,641 putative *Malassezia globosa* reads in sample h2540^28^. Nonetheless, when Ge et al. realigned the raw sequences of h2540 to the *M. globosa* reference genome^19^, they found that almost all of these supposed fungal reads actually mapped to two specific locations: an 897‐bp allele and a 557‐bp allele. Further BLAST analysis confirmed that both alleles were actually human sequences that were misidentified as *M. globosa*. This finding revealed the serious errors that could arise from reference genome contamination. Notably, the genome assembly of *M. globosa* was revised in December 2023, with NCBI removing five sequences that were determined to be contaminated, including these two alleles (human sequence contamination). This revision emphasized the importance of continuous updating and optimization of reference genome databases to ensure the accuracy of tumor microbiome research.

It is important to emphasize that these limitations do not imply that WGS or RNA‐seq is inherently unsuitable for tumor microbiome research^29^. When applied with rigorously curated databases, exhaustive host depletion using the most complete human reference genomes, and appropriate controls, these technologies remain valuable tools for detecting genuine microbial signals—as evidenced by the consistent and biologically plausible identification of HPV in cervical cancer and *H. pylori* in gastric cancer even under stringent analytical conditions. The challenge lies not in the sequencing technology itself, but in the particular difficulty of mining microbial signals from host‐dominated, low‐biomass data archives that were not originally designed for microbiome research. This distinction is consequential: It means that WGS and RNA‐seq can yield reliable tumor microbiome data, but only when the full analytical pipeline—from database construction to normalization—is built with the unique properties of microbial data explicitly in mind.

#### Reagent contamination in sequencing procedures

While high‐throughput sequencing technologies have enhanced our ability to characterize microbial communities, they have also made detection more susceptible to laboratory reagents and environmental contaminants. DNA extraction kits, PCR reagents, and even controlled laboratory environments can introduce contaminating sequences, such as plasmid DNA, primers, adapters, and vector sequences^30^. Such contaminating sequences may be misidentified as authentic biological signals, causing large numbers of sequencing reads to be incorrectly classified as bacteria, fungi, or other microorganisms^31^. This issue is particularly severe when studying low microbial biomass samples (such as blood, placenta, or tissues), as exogenous contaminating sequences can mask rare endogenous microbial sequences, significantly affecting the accuracy of results^32^. The study by Narunsky‐Haziza et al. provided a typical case showing how contamination can affect microbiome analysis results. In their analyzed sample h1948, *Trichosporon asahii* appeared to be the most abundant species with 656,503 reads. However, upon further alignment of these reads with the *Trichosporon* genome, it was discovered that 99.99% of the matching reads were concentrated within the same 80‐bp region, of which 58 bp were identical to the Illumina sequencing vector sequence. This finding demonstrated that these reads were actually contaminants introduced during the sequencing process rather than genuine microbial signals.

### Unreliability of machine learning conclusions due to Voom–SNM normalization

Significant technical heterogeneity exists across TCGA datasets, arising from differences in sequencing centers and platforms. To improve comparability when comparing across samples, data normalization is typically required. In Poore et al.'s study, the Voom–SNM normalization method was used to address this heterogeneity. Although the Voom method can transform heteroscedastic data into an approximately normal distribution, it was originally designed specifically for RNA sequencing data. For microbiome data with highly sparse characteristics, use of this algorithm may significantly affect data interpretability^33^. There are two main concerns with this approach. First, the distribution of tumor types across sequencing centers is uneven. For example, data from 10 tumor types, including TCGA–Adrenocortical Carcinoma (ACC) and TCGA–Cholangiocarcinoma (CHOL), came exclusively from the University of North Carolina sequencing center. As for the Washington University School of Medicine center, it only included four tumor types: TCGA–BRCA, TCGA–CESC, TCGA–Sarcoma (SARC), and TCGA–Uterine Corpus Endometrial Carcinoma (UCEC). By applying this normalization method to correct for batch variables such as sequencing centers, Poore et al. inadvertently introduced tumor type‐specific features into the data. Machine learning models could then pick up on these artificial signals, leading to predictions of tumor type based on microbial composition that appeared robust but were in fact unreliable. Second, Voom–SNM uses a combination of log transformation and supervised normalization to adjust the data, converting original zero values into non‐zero values^34^. In microbiome research, true zero values are common and biologically meaningful. Changing these values introduces spurious variations in the data, leading machine learning algorithms to make predictions based on variables that were originally zero, even though these variables should not have any influence on the predictions. In conclusion, the assumptions and transformations inherent in the Voom–SNM method, designed for RNA‐seq data, may not be well suited to the unique properties of sparse microbiome data. More domain‐aware normalization approaches tailored to the characteristics of microbiome data may be needed to address technical variation while preserving biological signals.

Why have these methodological flaws persisted in high‐profile studies? A root cause lies in a systemic cross‐disciplinary gap. Oncologists and bioinformaticians who work primarily with cancer genomics data often apply microbiome analysis pipelines without fully appreciating the unique ecological properties of microbial data—extreme sparsity, compositionality, and the pervasive low‐biomass challenge. Conversely, microbiologists with deep domain knowledge of these properties may lack the context of tumor heterogeneity and the structure of large cancer genomics repositories such as TCGA. The practical consequence is the uncritical transposition of RNA‐seq analytical tools—designed for a very different data structure—into microbiome workflows where their statistical assumptions do not hold. Voom–SNM normalization is a paradigmatic example: A method entirely appropriate for its intended purpose became a source of systematic artifacts when applied outside that domain. Addressing this gap requires not only better computational standards but also the cultivation of genuinely interdisciplinary research teams and community‐level consensus on what constitutes a rigorous tumor microbiome workflow.

## REFINED PROTOCOLS FOR TUMOR MICROBIOME ANALYSIS

### Optimization strategies for tumor microbial quantification workflow

These issues discussed above led to the retraction of Poore et al.'s article, further revealing systemic challenges in current tumor microbiome research. Based on recent research evidence and methodological advances^10^, ^19^, we propose an optimized tumor microbiome quantification workflow (Figure 1). For raw sequencing data derived from patient samples, host sequence depletion requires the latest high‐quality human reference genomes such as GRCh38 (hg38) or more complete reference genomes such as T2T–CHM13 and the pangenome^25^, ^35^. When processing transcriptome data, it is important to account for the influence of transcription on data processing. This requires an additional host depletion step, for which resources like the GENCODE reference database are ideal^36^. This step aims to minimize human genome sequence interference, enhancing the precision of downstream microbial quantification. Simultaneously, potential contamination sources need to be systematically eliminated. For microbial taxonomic classification, we recommend constructing and utilizing a comprehensive reference database. This database should include completed microbial genomes, plasmid genomes, vector genomes, phage genomes (such as the phiX174 virus genome), complete human reference genomes, and other genome sequences that may interfere with microbial classification. Additionally, we suggest using advanced bioinformatics tools such as Squeegee and decontam package for the identification and potential removal of contaminating sequences^37^. By implementing these optimization strategies, we expect to significantly improve the accuracy of tumor microbiome quantification while minimizing false‐positive rates.

**Figure 1:**
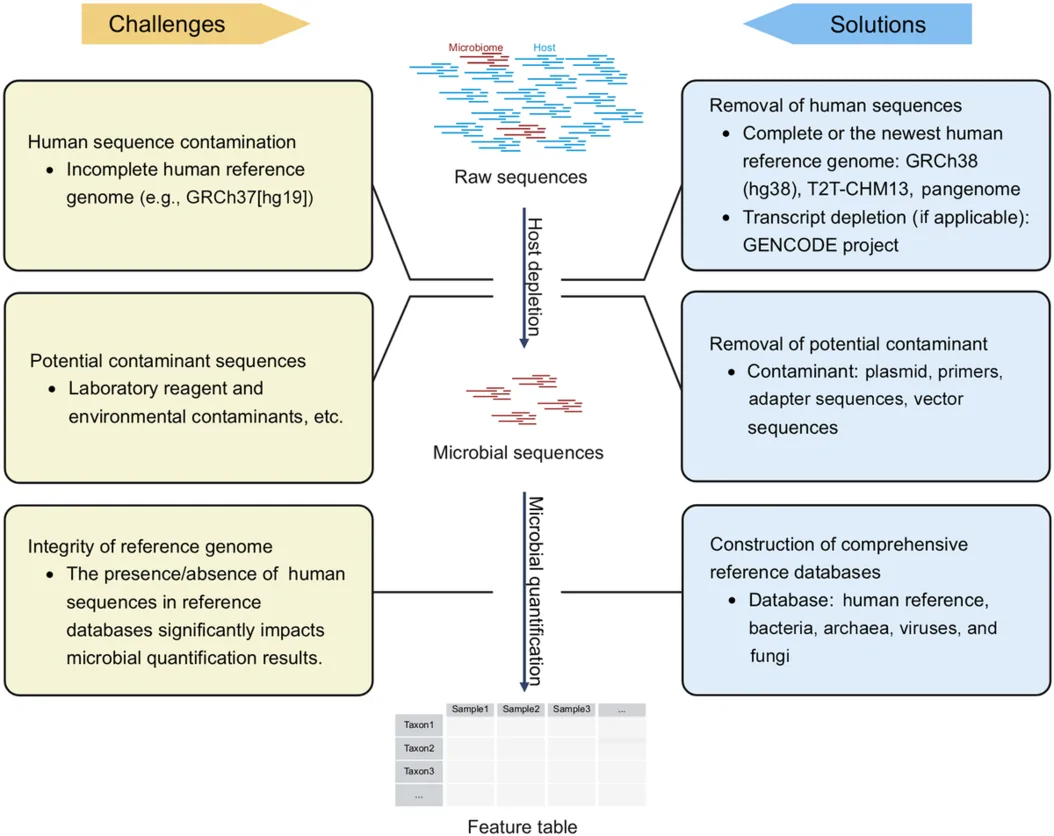
Methodological challenges and optimized strategies in tumor microbial quantification workflow. This schematic overview highlights major technical challenges in tumor microbiome analysis, including incomplete host depletion, potential contaminant sequences, and reference genome integrity issues, alongside corresponding solutions. These solutions include removal of human sequences using updated reference genomes, removal of potential contaminant sequences, and construction of comprehensive reference databases.

### Enhancing the robustness of microbial quantification

After obtaining microbial abundance information, several measures can help evaluate the reliability of quantified microbial abundances. By systematically analyzing genome coverage uniformity, we can effectively validate the microbial detection results, particularly in detecting potential interference from exogenous factors such as vector sequences. For instance, uneven genome coverage typically occurs when microbial identification stems from sequence misalignments or similarities with cloning vectors used in sequencing reagent production. Specifically, if all matching reads from a microorganism align only to a restricted region of its genome (usually spanning tens to hundreds of base pairs), this may indicate reagent or potential environmental contamination. In such cases, we suggest further analysis to determine whether these reads share high similarity with known vector sequences, plasmid sequences, or incompletely depleted human sequences. Furthermore, to improve the credibility of the results, we recommend aligning the reads identified as microbial against potential contamination sources, such as the latest human reference genome, to confirm that these sequences are genuine microbial reads. This step serves to exclude potential human sequence contamination, thereby strengthening the accuracy and credibility of microbial quantification results.

### Specific analytical methods for microbiome data

Microbiome data typically show highly sparse distribution patterns, differing fundamentally from transcriptomic data structures. This distinct characteristic demonstrates that many algorithms and tools developed for transcriptomic analysis may not be optimal for microbiome data processing and analysis, thus requiring the development of analytical strategies better suited for microbiome studies. Given the unique nature of microbiome data, specialized tools such as ConQuR and MMUPHin are recommended for batch effect correction^38^. These tools, specifically designed for microbiome data batch correction, effectively reduce feature‐specific biases while minimizing the risk of introducing spurious signals. In contrast, conventional transcriptomic analysis methods (such as Voom–SNM normalization) may introduce excessive artifactual signals when processing microbiome data^10^. Moreover, to further enhance the reliability of research findings, we recommend prioritizing further analysis of microorganisms that are consistently detected across multiple independent, rigorously decontaminated cohorts when conditions permit. This approach can substantially enhance the robustness and reproducibility of research discoveries.

## COMPREHENSIVE FRAMEWORK IN TUMOR MICROBIOME STUDIES

Tumor microbiome research faces numerous challenges, including inadequate host depletion in quantification workflows and suboptimal selection of analytical methods. These issues significantly impact the reliability, reproducibility, and robustness of research findings. Therefore, it is crucial to establish an integrated tumor microbiome analysis pipeline that encompasses sample collection, data analysis, causal inference, and experimental validation. Here, we propose a comprehensive research framework that integrates high‐throughput sequencing analysis with functional experiments to enable cross‐validation and complementarity across multidimensional data, thereby enhancing the reliability of research outcomes (Figure 2).

**Figure 2:**
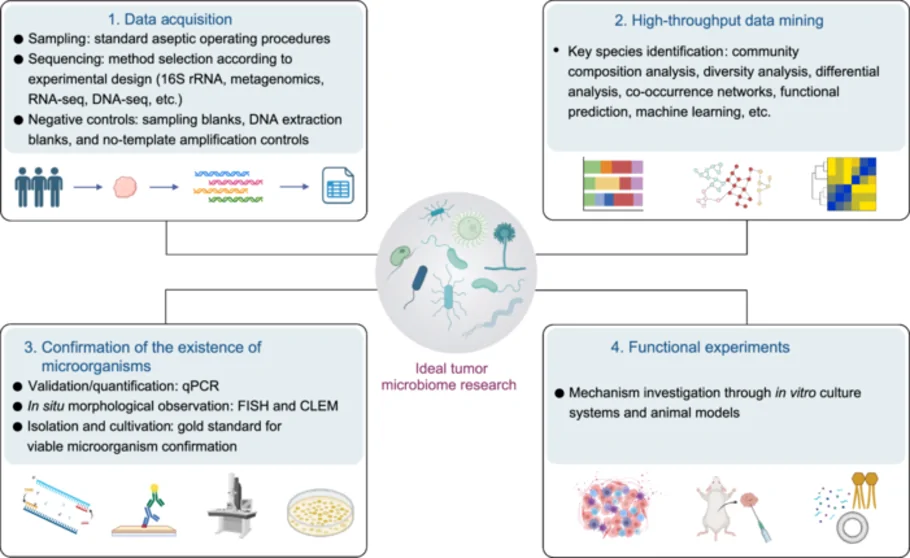
Proposed comprehensive research framework for rigorous tumor microbiome studies. The integrated workflow encompasses four key stages: (1) data acquisition, including aseptic sample collection, sequencing method selection, and multi‐level negative controls; (2) high‐throughput data mining, including microbial community profiling, diversity analysis, differential analysis, co‐occurrence network analysis, functional prediction, and machine learning; (3) confirmation of the existence of microorganisms through molecular quantification, *in situ* morphological observation, and microbial cultivation; and (4) functional experiments using *in vitro* culture systems and animal models to investigate the roles of microorganisms in tumor biology. 16S rRNA, 16S ribosomal RNA; CLEM, correlative light and electron microscopy; DNA‐seq, DNA sequencing; FISH, fluorescence *in situ* hybridization; qPCR, quantitative polymerase chain reaction; RNA‐seq, RNA sequencing.

### Data acquisition

In microbiome research, sample collection, as the initial step of the entire experimental process, plays a decisive role in ensuring data quality and result reliability. Researchers must adhere to several key principles to achieve this goal. Given that potential contamination risks during sampling can profoundly impact experimental conclusions, implementing strict aseptic sampling techniques and establishing multiple negative controls are crucial steps in ensuring experimental quality. First, sample collection should be performed in a thoroughly sterilized environment to minimize the risk of environmental contamination. Second, researchers should strictly follow aseptic protocols and use sterilized tools and containers for sample collection. The samples should then be promptly stored under predetermined optimal conditions to maintain the integrity of microbial communities. For low‐biomass samples (such as tumor biopsy tissues), additional contamination prevention measures are necessary to ensure that external contamination does not mask or interfere with the already weak microbial signals within the tissue. This may include using specialized sampling tools and more stringent aseptic procedures. Meanwhile, comprehensive metadata should be recorded, including but not limited to collection time, location, method, patient clinical information, and any environmental or host factors that might affect microbial composition^39^. This information is crucial for subsequent data analysis and result interpretation. In addition, the experimental design should include multi‐level negative controls, such as sampling blanks, DNA extraction blanks, and no‐template amplification controls. These control samples effectively evaluate potential contamination introduced at various experimental stages and serve as important references for subsequent data filtering and quality control. Ideally, corresponding negative controls should be set up for each sampling environment and experimental batch. This systematic control setup can effectively identify and quantify specific contamination sources during the experimental process, providing a basis for further bioinformatic analysis to accurately filter potential contaminating sequences. In conclusion, these rigorous sample collection and quality control measures not only significantly reduce the risk of technical bias and false‐positive findings but also substantially enhance the interpretability, reproducibility, and credibility of research results, laying a solid foundation for clinical translation.

The rapid development of high‐throughput sequencing technologies has brought transformative advances to microbiological research. Compared to traditional single‐strain isolation and culture methods, these technologies enable simultaneous screening and analysis of large numbers of microorganisms. This allows researchers to rapidly and comprehensively characterize the composition and function of complex microbial communities. Among various high‐throughput sequencing methods, 16S rRNA sequencing and metagenomic sequencing have emerged as the two most widely used techniques in microbial research, each with its unique advantages^40^. 16S rRNA sequencing is widely used in large‐scale microbiome studies and preliminary investigations due to its cost‐effectiveness and ability to avoid host DNA interference. This technique effectively reduces interference from human sequences in low‐biomass samples (such as tumor tissue samples), but its main limitations lie in limited species resolution and lack of functional information^41^. In contrast, metagenomic sequencing provides higher taxonomic resolution and rich functional information, although it typically requires greater investment and is more vulnerable to host DNA contamination. When applied to low‐biomass samples, it requires appropriate host sequence depletion pipelines to minimize the impact of human sequences, or alternatively, increased sequencing depth and microbial enrichment to enhance the detection of low‐abundance microorganisms^42^. Consequently, 16S rRNA sequencing can be used to obtain a quick and economical overview of the microbial community in samples, while metagenomic sequencing enables in‐depth analysis of specific microorganisms or functions. Given that 16S rRNA sequencing and metagenomic sequencing each have their own advantages and limitations, combining both technologies can more accurately reveal microbial community structure and function. This combined strategy has been successfully applied in multiple studies, showing promising results, particularly in analyzing complex environmental samples and low‐biomass samples^43^.

In recent years, methods for quantifying microorganisms based on DNA and RNA sequencing data have gained increasing attention. With a scientifically and methodologically sound research design, these methods can maximize the extraction of microbial information from existing omics data^16^. However, as mentioned earlier, despite the merits of this analytical approach, a key challenge remains: how to accurately extract hidden microbial information from human tissue sample sequencing results through bioinformatic analysis methods. Due to the extremely low microbial biomass in tumor microenvironments, it is difficult to distinguish between genuine observations and contaminants^44^. Drawing on integrated evidence from multiple studies, we present a refined pipeline for tumor microbiome analysis that enhances both comprehensiveness and robustness (Figure 2). Researchers should select the most appropriate sequencing technology based on the research objectives, sample characteristics, and anticipated microbial abundance.

### High‐throughput data mining

Following sequencing and quality control, comprehensive bioinformatic analyses are essential for characterizing tumor‐associated microbial communities. Taxonomic profiling can be used to assess microbial composition and abundance, while alpha‐ and beta‐diversity analyses characterize within‐sample diversity and between‐sample community differences, respectively. Differential abundance analysis may identify microbial taxa associated with specific tumor characteristics or clinical outcomes. Co‐occurrence network analysis can further reveal potential associations among microbial taxa. Functional prediction and pathway analysis can provide insights into the potential metabolic capabilities of microbial communities and generate hypotheses for subsequent experimental validation. In addition, machine learning approaches may facilitate the identification of microbial biomarkers and the development of predictive models. Independent validation is also important for ensuring the robustness and reproducibility of identified microbial signatures.

### Confirmation of the existence of microorganisms

Defining a “true” intratumoral microbial signal requires meeting strict, multi‐modal criteria. Although high‐throughput sequencing technologies can provide comprehensive and detailed microbiome landscapes, they are susceptible to batch effects, and nonspecific amplification in low‐biomass samples, potentially leading to false‐positive results and affecting data reliability and accuracy^45^. We propose that a signal should be considered validated only when it fulfills criteria across four complementary dimensions, each addressed by a distinct class of experimental method. First, statistical decontamination: decontamination algorithms such as decontam, validated using multiple negative controls, should confirm that a taxon cannot be explained by reagent or environmental contamination. Second, physical presence: qPCR, fluorescence *in situ* hybridization (FISH), and correlative light and electron microscopy (CLEM) should confirm that the organism is physically present in the tissue. qPCR, with its high sensitivity and specificity, not only validates the sequencing results but also enables precise quantification of specific microorganisms. By using appropriate negative controls, such as no‐template controls and human genomic DNA negative controls, nonspecific amplification can be effectively eliminated, thereby improving result reliability. FISH and CLEM technologies enable direct visualization and precise localization of specific microorganisms in tissue samples. These techniques provide information about the spatial distribution of microorganisms in host tissues and reveal microbe–host cell interactions. They represent valuable tools for *in situ* morphological observation. Third, culture‐based methods remain the gold standard for confirming that detected microorganisms are viable rather than nucleic acid debris^46^ and for providing essential material for subsequent functional studies. Fourth, functional relevance: Experimental models should demonstrate a causal link between microbial presence and a measurable biological effect, as discussed in the "functional experiments" section below. Only by satisfying all four dimensions can a microbial signal be considered a credible candidate for biological and clinical investigation. In the study by Nejman et al., researchers used a comprehensive set of methods, not only validating the presence of bacteria in various tumor types but also revealing the complexity and heterogeneity of tumor microbiomes. These findings provide important evidence for understanding the role of microorganisms in tumor development^47^.

### Functional experiments

To advance tumor microbiome research from correlative description to causal explanation, functional experiments are indispensable. Sequencing‐based profiling identifies which microorganisms are present and in what abundance, but cannot on its own establish whether they drive, modify, or are merely passengers within the tumor microenvironment^48^. A range of experimental models is available, each suited to answering distinct mechanistic questions. Germ‐free mouse models, colonized with specific bacterial isolates, provide the most direct *in vivo* evidence of causality, enabling researchers to observe tumor initiation, growth, immune modulation, and metastatic behavior in the presence or absence of defined microbial species under controlled conditions. Conventional mouse models with antibiotic depletion, fecal microbiota transplantation, or direct intratumoral injection of specific microorganisms into subcutaneous tumor xenografts offer a complementary approach, allowing modulation of the tumor‐associated microbiome in immunocompetent hosts to assess the net impact of microbial communities on tumor progression and treatment response^49^. Patient‐derived organoid co‐culture systems provide a clinically relevant *in vitro* platform for dissecting direct interactions between specific bacterial strains and tumor cells. These systems capture effects on proliferation, invasion, drug sensitivity, and immune evasion while preserving the three‐dimensional architecture and genetic heterogeneity of the original tumor. Transwell and direct co‐culture assays with isolated bacterial strains or conditioned media can further isolate the contributions of secreted metabolites, structural components, or contact‐dependent signaling to specific cellular phenotypes^50^. Together, these models form a complementary toolkit that, when applied systematically, can establish the causal role of intratumoral microorganisms and elucidate the mechanisms through which they influence tumor biology.

## CONCLUDING REMARKS

Here, we systematically summarize the major challenges and methodological limitations in current tumor microbiome research, and examine methodological considerations in Poore et al.'s study and their impact on the entire field. Through a comprehensive review and critical analysis of the existing literature, we propose a series of practical optimization strategies for analytical workflows to enhance the reliability, reproducibility, and clinical relevance of tumor microbiome studies. These strategies encompass the entire pipeline from sample collection and high‐throughput sequencing to data analysis and functional validation, providing comprehensive methodological guidance for future research while laying the foundation for clinical applications. Although tumor microbiome research still faces ongoing controversies and technical challenges, the close association between intratumoral microorganisms and tumor biology, along with their potential clinical significance, has become increasingly evident. Advances in this field have provided new avenues for cancer diagnosis and treatment. With advancing high‐throughput sequencing technologies and continuous optimization of bioinformatic methods, we are better equipped to reveal the complex microbial community structures and their functions within the tumor microenvironment. However, the retraction of Poore et al.'s article serves as a reminder of the crucial importance of maintaining scientific rigor and critical thinking in this emerging and complex field. It also highlights the necessity of establishing more stringent analytical workflows.

To advance tumor microbiome research, we call on researchers to optimize and standardize quantitative analytical workflows and to establish internationally recognized consensus guidelines. Specifically, several key aspects deserve particular attention. First, rigorous control of contamination during sample collection and processing must be implemented, incorporating adequate negative controls. Second, appropriate sequencing strategies should be selected and bioinformatic pipelines optimized, with particular attention paid to host sequence depletion efficiency and reference database comprehensiveness. Third, sequencing results warrant validation using complementary experimental techniques, including qPCR, FISH, and microbial cultivation. Finally, carefully designed functional experiments are required to elucidate the causal relationships and underlying mechanisms linking microorganisms to tumorigenesis.

Looking ahead, with continuous innovation and refinement in research methodologies, particularly the advancement of multi‐omics integrated analysis and functional experimental validation, coupled with the application of artificial intelligence and machine learning in data analysis, tumor microbiome research is expected to advance in several aspects: (1) more precise mapping of microbial profiles in different types of tumors, thereby revealing the relationships between microbial communities and tumor progression; (2) elucidation of the molecular mechanisms by which microorganisms modulate tumor development through regulation of host immunity and metabolism; (3) development of microbiome‐based biomarkers for early cancer diagnosis and prognosis prediction; (4) and exploration of novel cancer treatment strategies targeting the microbiome, such as microbial vaccines and probiotic interventions.

In conclusion, despite numerous challenges, growing evidence points to the existence of the intratumoral microbiome and its impact on the host, although rigorous validation is still urgently needed before any clinical translation can be considered. It holds promise as a new frontier in cancer research. As research methods improve and our understanding of microbe–tumor interactions deepens, tumor microbiome research promises to yield new insights into the molecular mechanisms of tumor initiation, progression, and metastasis. It will advance the development of more precise cancer diagnosis and personalized treatment strategies, ultimately bringing new hope to cancer patients.
